# High-resolution melting (HRM) re-analysis of a polyposis patients cohort reveals previously undetected heterozygous and mosaic *APC* gene mutations

**DOI:** 10.1007/s10689-015-9780-5

**Published:** 2015-01-21

**Authors:** Astrid A. Out, Ivonne J. H. M. van Minderhout, Nienke van der Stoep, Lysette S. R. van Bommel, Irma Kluijt, Cora Aalfs, Marsha Voorendt, Rolf H. A. M. Vossen, Maartje Nielsen, Hans F. A. Vasen, Hans Morreau, Peter Devilee, Carli M. J. Tops, Frederik J. Hes

**Affiliations:** 1 Department of Clinical Genetics, Leiden University Medical Center, P.O. Box 9600, 2300 RC Leiden, The Netherlands; 2 Department of Clinical Genetics, Academic Medical Center, Amsterdam, The Netherlands; 3 Department of Clinical Genetics, University Medical Center Nijmegen, Nijmegen, The Netherlands; 4 Department of Human Genetics, Leiden University Medical Center, Leiden, The Netherlands; 5 Department of Gastroenterology, Leiden University Medical Center, Leiden, The Netherlands; 6 The Netherlands Foundation for the Detection of Hereditary Tumours, Leiden, The Netherlands; 7 Department of Pathology, Leiden University Medical Center, Leiden, The Netherlands

**Keywords:** APC, High-resolution melting, Mosaicism, Polyposis, Colorectal cancer, Molecular diagnostics

## Abstract

Familial adenomatous polyposis is most frequently caused by pathogenic variants in either the *APC* gene or the *MUTYH* gene. The detection rate of pathogenic variants depends on the severity of the phenotype and sensitivity of the screening method, including sensitivity for mosaic variants. For 171 patients with multiple colorectal polyps without previously detectable pathogenic variant, *APC* was reanalyzed in leukocyte DNA by one uniform technique: high-resolution melting (HRM) analysis. Serial dilution of heterozygous DNA resulted in a lowest detectable allelic fraction of 6 % for the majority of variants. HRM analysis and subsequent sequencing detected pathogenic fully heterozygous *APC* variants in 10 (6 %) of the patients and pathogenic mosaic variants in 2 (1 %). All these variants were previously missed by various conventional scanning methods. In parallel, HRM *APC* scanning was applied to DNA isolated from polyp tissue of two additional patients with apparently sporadic polyposis and without detectable pathogenic *APC* variant in leukocyte DNA. In both patients a pathogenic mosaic *APC* variant was present in multiple polyps. The detection of pathogenic *APC* variants in 7 % of the patients, including mosaics, illustrates the usefulness of a complete *APC* gene reanalysis of previously tested patients, by a supplementary scanning method. HRM is a sensitive and fast pre-screening method for reliable detection of heterozygous and mosaic variants, which can be applied to leukocyte and polyp derived DNA.

**Electronic supplementary material:**

The online version of this article (doi:10.1007/s10689-015-9780-5) contains supplementary material, which is available to authorized users.

## Introduction

Familial adenomatous polyposis (FAP) is a hereditary tumor syndrome predisposing to early-onset colorectal cancer (CRC), accounting for 1 % of CRC cases. Classic FAP patients develop well over a hundred of colorectal polyps starting from early adolescence, leading to CRC approximately a decade later. Attenuated FAP (AFAP) patients develop 10–100 polyps at an older age at onset in their forties [1, 2]. Most FAP and AFAP patients carry dominantly inherited pathogenic germline variants in the *APC* gene (MIM: 175100) or recessive germline variants in the *MUTYH* gene (MIM: 604933). Pathogenic *APC* or *MUTYH* variants can also cause extracolonic features, like duodenal and gastric adenomas and cancer, desmoid tumors, osteomas and benign skin tumors (Gardner syndrome).

The detection rate of pathogenic *APC* variants, including large structural variation, is approximately 70–80 % in classic FAP patients and 10–30 % in AFAP patients [3–6]. The *MUTYH* associated polyposis (MAP) phenotype generally resembles AFAP. Bi-allelic pathogenic *MUTYH* variants are found in a quarter of polyposis patients negative for pathogenic *APC* variants [7]. In 20–30 % of adenomatous polyposis patients, no pathogenic variant in *APC* or *MUTYH* is identifiable, especially in patients with low polyp counts [7, 8]. A few polyposis patients have been linked to other genes, such as *SMAD4*, *BMPR1A*, *POLE* and *POLD1* [8, 9]. Pathogenic variants in unexplored genes may underlie the remaining genetically unexplained polyposis patients. However, two considerations warrant further examination of the *APC* gene, with its paramount role in the mechanism of polyposis and somatic defects in almost all adenomatous polyps.

First, in the past 20 years, a variety of methods have been applied to identify pathogenic germline *APC* variants, including direct sequencing and indirect methods like the protein truncation test (PTT), denaturing high performance liquid chromatography (dHPLC) and denaturing gradient gel electrophoresis (DGGE), southern blot and multiplex ligation-dependent probe amplification (MLPA). The detection rate of pathogenic variants is partly determined by the sensitivity of these methods and to which extend the length of the *APC* gene was tested [10, 11]. Second, mosaic pathogenic *APC* variants are particularly difficult to detect and probably an underestimated cause of polyposis coli. Of all detected constitutive pathogenic *APC* variants, 15–25 % occur de novo and 1–4 % are mosaics [12, 13].

In this study a reanalysis of the entire coding region of *APC* was performed in a group of 171 patients with ≥10 colorectal polyps, without previously detectable pathogenic *APC* (or *MUTYH*) variants. *APC* scanning had been performed before by various methods with different levels of sensitivity and completeness [12]. To identify possible previously missed heterozygous and mosaic variants, we used one uniform technique: high-resolution melting (HRM) analysis, which is known to be able to reliably detect heterozygous variants and lower allelic fractions [14, 15]. Also, scanning in polyp DNA of two patients was performed, as a proof of principle, to identify possible mosaic variants.

## Materials and methods

### Patient samples

The study group consisted of 171 index patients, referred for *APC* and/or *MUTYH* gene testing at the Laboratory for Diagnostic Genome Analysis (LDGA) in Leiden, The Netherlands, between 1995 and 2007, without detectable pathogenic variants by analysis as earlier described [12]. Of the 244 consecutively referred patients 171 were included, diagnosed with ≥10 colorectal polyps of adenomatous or predominantly adenomatous histology. The excluded 73 patients had insufficient DNA quality (3), insufficient clinical data (13) or <10 polyps (57). Informed consent was obtained for DNA testing according to protocols approved by local ethics review board. Clinical data was obtained from patient records at the LDGA (Table 1). DNA was extracted from blood leukocytes according to standard protocols. Tissue DNA was analyzed if applicable and available for part of the patients. For HRM validation in total 117 DNA samples with unique heterozygous variants and for each amplicon 10 wild-type samples available at the LDGA were used. Additionally homozygous, heterozygous and wild-type controls of eight common SNPs were tested.

**Table 1:** Patient characteristics

Clinical characteristics	Mean (range)	n	Pathogenic *APC* variant
Gender			
Male		96	
Female		75	
Age diagnosis polyps	50 (14–73)		
Polyp number			
10–29	53 (20–69)	56	1
30–99	55 (30–73)	45	1
>100	43 (33–70)	14	2
‘Multiple/Polyps/Ten’s/Polyposis’	46 (14–67)	56	6 + 2 mosaics
CRC			
Yes		63	1 + 1 mosaic
No		108	9 + 1 mosaic
Family history 1st and 2nd degree			
Polyps (with or without CRC)		60	6
Parents		21	3
Sibs		47	5
Grandparents		1	
Offspring		2	
CRC, no polyps		36	3 + 1 mosaic
No polyps and CRC		62	2 + 1 mosaic
Unknown		13	1

### PCR, primers and unlabeled probes

The *APC* gene is located at 5q22.2, spans 163,719 bp, contains 15 coding exons with an open reading frame of 8,532 bp and encodes 2,843 amino acids (OMIM: 611731, Genbank: NG_008481.4, NM_000038.5, http://www.ncbi.nlm.nih.gov/nuccore). Primers were designed with Lightscanner Primer Design Software (Idaho Technology, Salt Lake City, UT) and comprised M13 sequencing tails. *APC* exons 1–15 were covered by 61 amplicons, of which 42 in exon 15, including exon–intron boundaries, but not the untranslated regions (5′ and 3′ UTRs). Amplicon length was chosen approximately 150–300 bp [16]. For exons covered by multiple amplicons, the minimal overlap between amlicons was 30 bp, excluding primer regions. PCR conditions were as previously described [16–18]. Unlabeled probes were designed for eight amplicons with common SNPs (5, 11, 13, 15.18, 15.22, 15.23, 15.24 and 15.27), complement to the wild-type strand, with a length of ~30 bp, melting temperature of <70 °C and GC content between 40 and 45 % [16, 17] (online resource Supp. Table S1).

The 10 µl PCR mix contained 20 ng of template DNA, 1× Lightscanner Mastermix (including LC Green Plus dye, Idaho Technology), 2.5 pmol of each primer and distilled water. In our hands results with Lightscanner Mastermix were better compared to separately mixed ingredients (data not shown). Template DNA concentrations were measured by a Nanodrop 1000 Spectrophotometer (Thermo Fisher Scientific, Wilmington, DE). Asymmetric PCRs with unlabeled probes were performed with a 1:5 ratio, with 1 pmol of forward primer, 5 pmol of reverse primer and 5 pmol of the unlabeled probe. Optimal primer annealing temperatures were established by temperature gradient PCR and HRM. Amplicons were redesigned if they showed more than two melting domains [17] (online resource Supp. Fig. S1).

### HRM data analysis

Melting of PCR products was performed on a 96 wells Lightscanner instrument, with Lightscanner software version 2.0 (Idaho Technology). Temperature ranges of 70–98 and 55–98 °C were used for amplicon scanning and unlabeled probe genotyping, respectively, with increments of 0.1 °C/s. Melting curve data analysis was performed as described [16, 17]. The software’s sensitivity level was set to ≥3.0. Raw melting curves were normalized at 100 and 0 % fluorescence intensity. They were also temperature shifted, at 5 or 95 % fluorescence intensity, to correct for slight temperature differences across the plate. Melting analysis was performed twice per PCR plate to rule out melting artifacts.

### Variant scanning and sequencing

PCR and HRM were performed in 96 well plates, including positive controls per amplicon in each plate, with allelic fractions of 50, 13 and 6 %. PCR and HRM were repeated if the first experiment showed an aberrant melting curve. Sequencing was performed if the repeated experiment showed an aberrant melting curve, except for those that were induced by a SNP under a specific probe. The overall portion of succeeded tests, including HRM and sequencing per amplicon, for the 171 patients × 61 amplicons was 99 %. Direct sequencing was performed as described, directly after HRM, on the same reaction mix and analyzed by Seqscape software version 2.5 [12, 17]. Sequences were carefully observed for detection of low peaks. Pyrosequencing was performed as described, to confirm low-level mosaic variants [12]. Detected variants were searched in literature and the UMD and LOVD public databases (www.lovd.nl/apc, www.umd.be/apc, [19–22]). New variants were analyzed in silico for possible pathogenicity by Alamut version 2.3 (Interactive Biosoftware, Rouen, France).

## Results

### HRM variant scanning in patients with multiple polyps

#### Pathogenic heterozygous variants

In the 171 scanned patients, HRM analysis and subsequent sequencing detected eight different heterozygous pathogenic variants occurring in ten patients (6 %). Four of the pathogenic variants were novel and four were previously reported in the literature. Three novel pathogenic variants were frameshifting variants and one was a duplication at the splice donor site of intron 14, (c.1958 + 1_1958 + 2dup, patient 7). In silico analysis by Alamut predicted an in frame skip of exon 14, which was confirmed by reverse transcriptase PCR (data not shown). In nine out of the ten patients with a pathogenic variant, it had been missed by previous diagnostic testing methods, respectively PTT at the 5′ part of exon 15, DGGE in exons 9, 14 and 15 or single-strand conformational polymorphism (SSCP) in exons 9 and 11. One pathogenic variant was located in a region of the gene not tested in that particular patient before (Table 2, online resource Supp. Table S2).

**Table 2:** Pathogenic variants and two VUS detected and corresponding clinical data in the 171 patients^a, b^

Amplicon	Variant DNA (allelic %)	Variant protein	No.	Sex	No of polyps (age in years)	CRC, other (age in years)	Family history^c^
*Pathogenic*							
6	c.679del	p.Asp227Thrfs*66	1	F	>100 (43)	–	P
9.3	c.1180C>T	p.Gln394*	2	M	Polyposis (48)	–	P, C
			3	F	>20 (58)	–	P, C
			4	M	70 (57)	–	P, C
9.3	c.1248C>A	p.Tyr416*	5	F	Polyposis (27)	Papillary thyroid carcinoma (19)	P
11	c.1548 + 1G>A	p.spl	6	M	Polyposis (48)	CRC (48)	No
14.2	c.1958 + 1_1958 + 2dup	p.spl	7	M	Polyposis (56)	Gastric cancer	P
15.1	c.1972_1975del	p.Glu658Thrfs*11	8	F	Multiple (41)	–	Unknown
15.1	c.2003del	p.His668Profs*2	9	M	10 s (32)	–	P, C
15.2	c.2222dup	p.Asn741Lysfs*15	10	M	>100 (16)	–	C
*Mosaic*							
15.3	c.2269C>T (~ 5 %)	p.Gln757*	11	M	Countless adenomas (36)	Duodenal adenomas (37)	No
15.17	c.4393_4394dup (~15 %)	p.Ser1465Argfs*9	12	M	Multiple (44)	CRC (50), Gastric and duodenal polyps	No
*Polyp tissue only*							
15.14	c.4057G>T	p.Glu1353*	13	V	32 (17); only in rectosigmoid	–	No
*Polyp tissue only (outside study group)* ^b^							
15.19/20	c.4666dup	p.Thr1556Asnfs*3	14	M	>100 (26)	–	Unknown
*VUS*							
15.24	c.5501_5506del	p.Val1834_Arg1835del	15	M	100	–	P, C^d^
15.29/30	c.6363_6365dup	p.Ala2122dup	16	F	>13 (58)	–	C^e^

^a^Pathogenic variants and a selection of two VUS are shown (an in-frame deletion and an in-frame insertion). The other VUS and SNPs (common and rare missense and silent variants) are shown in online resource Supp. Table S2. cDNA nomenclature is according to NCBI Reference sequence NM_000038.5

^b^Of two patients DNA isolated from polyps was available for HRM of *APC*. Patient 13 was part of the study group of 171 patients referred between 1995 and 2007 and patient 14 was referred after this interval in 2008

^c^Family history in 1st and 2nd degree relatives. Only polyps or CRC are given. P: polyps, C: CRC

^d^The c.5501_5506del variant was present in the son (not affected at age 40 years) and the sister (CRC and polyps) of the index patient. The son of the sister (multiple polyps at age 40 years) did not carry the VUS

^e^For patient 16 no family members were tested

#### VUS and polymorphisms

Sixteen different rare variants of unknown significance (VUS) were found in 17 of the 171 scanned patients, of which five were novel, including one silent variant, two missense variants, one in frame deletion and one in frame duplication. No effects on splicing or other in silico clues for pathogenicity were predicted by Alamut. All of these VUS were found in patients without a pathogenic variant, except one (online resource Supp. Table S2). The in-frame deletion c.5501_5506del, p.Val1834_Arg1835del was found in a patient with 100 colorectal polyps (patient 15). Segregation of the variant with the disease in this family suggests that causal relevance of this VUS as a high-penetrant pathogenic variant is unlikely (see Table 2). The in frame duplication c.6363_6365dup, p.Ala2122dup was found in a patient with >13 polyps (patient 16), for whom no further family members were available for segregation analysis (Table 2, online resource Supp. Table S2). One known VUS was the Ashkenazim low risk variants c.3920T>A, p.Ile1307Lys, detected in one patient, of which the allele frequency in the scanned group of 171 patients was similar to Caucasian dbSNP populations (online resource Supp. Table S2) [23].

#### Pathogenic mosaic variants

**Fig. 1:**
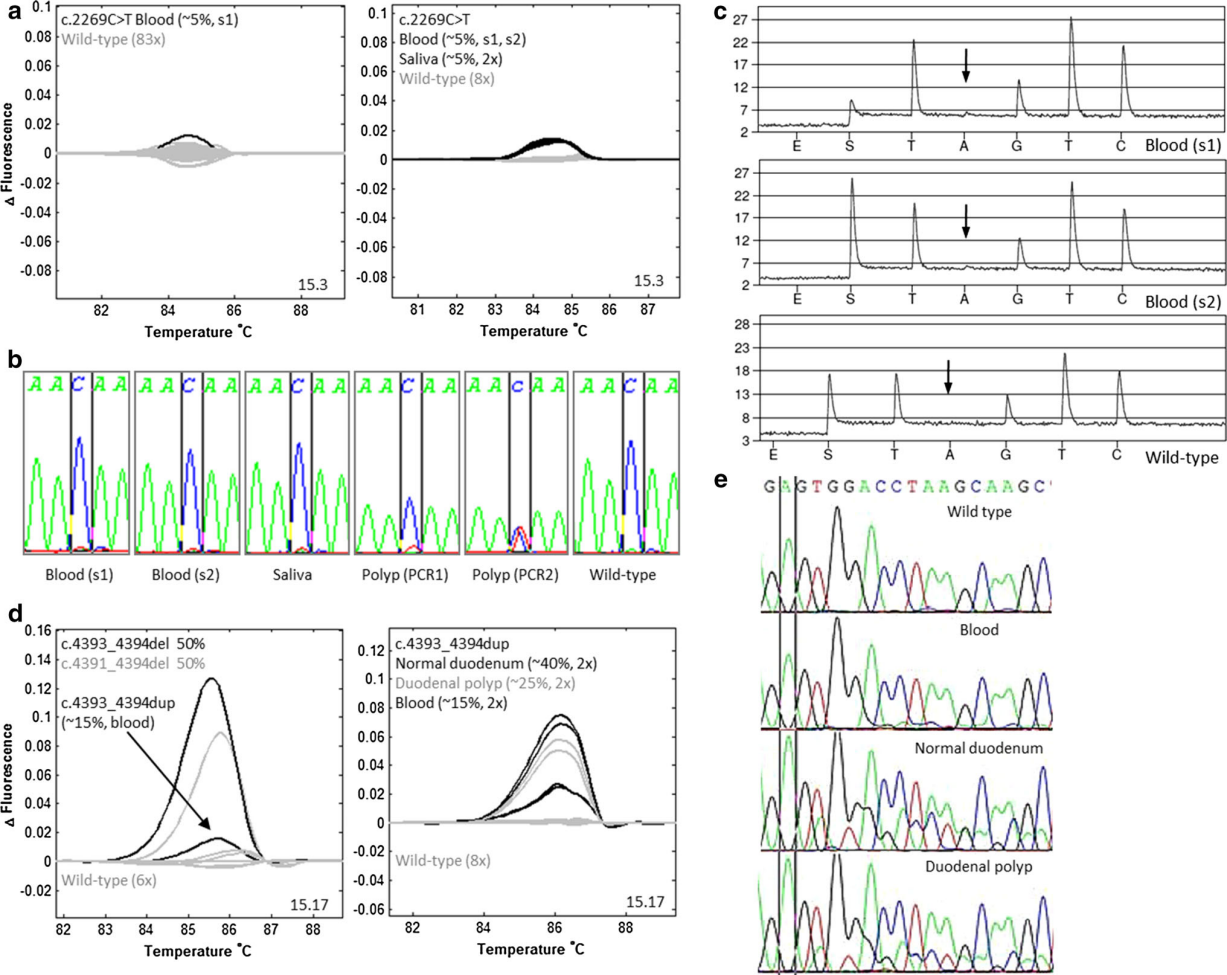
Pathogenic mosaic variants in leukocyte and tissue DNA from patients 11 and 12. **a** Patient 11, HRM: a minimally aberrant curve in amplicon 15.3 in repeated experiments. Blood and saliva show a comparable allelic fraction. HRM of polyp DNA failed (data not shown). **b** Patient 11, direct sequencing: reverse sequence with a c.2269C>T mosaic variant, a very small T-peak in blood and saliva (~5 % allelic fraction), not visible in wild-type, and enriched in one polyp (~20–50 % allelic fraction). Different PCR reactions showed a different result in the polyp, possibly due to preferential amplification. **c** Patient 11, pyrosequencing: reverse complement sequence with a very small A-peak, not visible in wild-type, calculated at an allelic fraction of 5 %. **d** Patient 12, HRM: a slightly aberrant curve in amplicon 15.17 in repeated experiments. As comparison curves of two control samples with heterozygous variants at the same location are shown. HRM showed enrichment in a duodenal polyp and in normal duodenal mucosa. **e** Patient 12, direct sequencing: forward sequence with a c.4393_4394dup mosaic variant, with an allelic fraction of ~15 %. Enrichment was visible in normal duodenal mucosa (~40 %) and the duodenal polyp (~25 %) [Color figure can be viewed in the online issue, which is available at http://link.springer.com/]

In two patients (1 %) pathogenic mosaic variants were detected in leukocyte derived DNA (patients 11 and 12). Patient 11 carried the pathogenic variant c.2269C>T, p.Gln757*, which was confirmed by pyrosequencing, and had an allelic fraction of 5 %. The allelic fraction in a saliva sample was also estimated at 5 % and in one tested colorectal adenoma it was enriched to 20–50 %. Patient 12 carried c.4393_4394dup, p.Ser1465Argfs*9 at an estimated allelic fraction of ~15 %. In a duodenal adenoma the allelic fraction was ~25 % and in normal duodenal mucosa ~40 %. The two pathogenic mosaic variants were missed in leukocyte derived DNA by previous diagnostic testing by DGGE and PTT and by PTT and sequencing, respectively (Table 2; Fig. 1, online resource Supp. Table S2).

#### Pilot scanning study to detect mosaic variants in polyp derived DNA in two patients

**Fig. 2:**
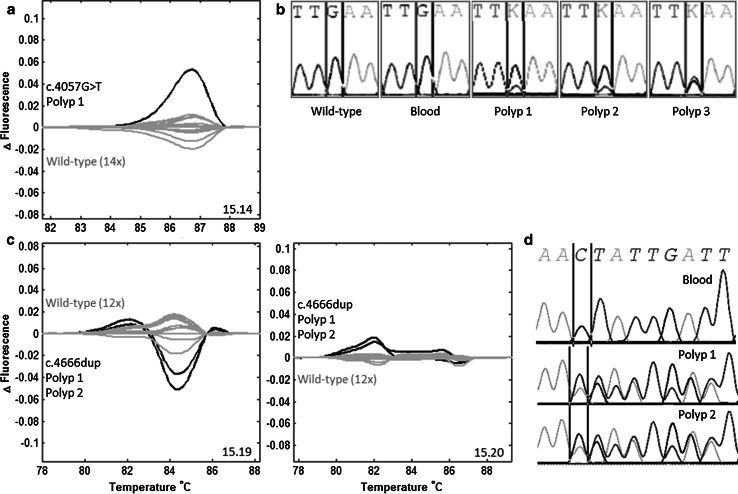
Pathogenic mosaic variants in tissue DNA, but not in leukocyte DNA from patients 13 and 14. **a** Patient 13, HRM: an aberrant curve from polyp DNA in amplicon 15.14 in repeated experiments. **b** Patient 13, direct sequencing: forward sequence from DNA samples isolated from three different polyps showing a c.4057G>T variant with an allelic fraction estimated at ~20–50 % in the sequence trace. This variant was not detectable in blood by sequencing (**b**) and HRM (data not shown). **c** Patient 14, HRM: an aberrant curve in overlapping amplicons 15.19 and 15.20 in repeated experiments, from DNA samples isolated from two different polyps, with different histology, adenomatous and hyperplastic. **d** Patient 14, direct sequencing: forward sequence showing a c.4666dup variant in both polyps, with an estimated allelic fraction of ~30 %. This variant was not detectable in blood, by sequencing (**d**) and HRM (data not shown) [Color figure can be viewed in the online issue, which is available at http://link.springer.com/]

The HRM set-up was tested on polyp-derived DNA of two apparently sporadic polyposis patients of whom tumor tissue was available (patients 13 and 14), and in whom no pathogenic *APC* variants in leukocyte-derived DNA were detected by HRM. Patient 13 was part of the study group. She had 32 adenomas limited to the rectosigmoid, diagnosed at the age of 17 years, without family history. In the DNA of three polyps the c.4057G>T, p.Glu1353* variant was found with an allelic fraction of 20–50 %. The variant was not detectable by HRM analysis and direct sequencing in cultured skin fibroblasts, buccal mucosa and urine. Patient 14 was referred for diagnostic *APC* testing after the time interval of the study group. He had >100 polyps diagnosed at the age of 26 years, without family history. The majority of polyps had adenomatous and a minority hyperplastic histology. In the DNA of one adenomatous and one hyperplastic polyp the c.4666dup, p.Thr1556Asnfs*3 variant was found, both with an estimated allelic fraction of ~30 % (Table 2; Fig. 2, online resource Supp. Table S2).

### HRM validation

#### Heterozygous variants

Amplicons were tested for sensitivity of the detection of heterozygous variants using the available positive control samples. Of the 117 tested samples with unique heterozygous variants in 50 of the 61 amplicons, 116 were detectable (online resource Supp. Fig. S2). The variant c.423-17dup, a duplication of a T in a T^7^A^13^-stretch in intron 3, was not detectable by HRM. This region has been described as problematic before [24]. Ten wild-type samples were tested per amplicon in duplicate, among which no false positives were seen.

#### Low allelic fractions

The 116 samples with detectable heterozygous variants were serially diluted to allelic fractions of 25, 13 and 6 % in a wild-type background. All 116 variants were detectable down to at least 13 % and 105 (90 %) down to at least 6 %. For 72 variants the dilutions were continued down to 3 and 2 % and 28 and 6 variants were detectable, respectively. The eleven variants not detectable below 13 % were eight 1-bp deletions or insertions, two base-pair neutral changes (A>T and T>A) and one A>G change (online resource Supp. Table S3). Of six variants tested in multiple overlapping amplicons, three showed different detection limits (6 vs. 25 % for one, and 3 vs. 6 % for two variants). Four variants were better detectable with temperature shift set at level 95 % instead of the default 5 % (online resource Supp. Fig. S2 and S3).

#### Common SNPs and unlabeled probes

Eight amplicons contained a common SNP, with a MAF >10 %, for which unlabeled probes were designed to facilitate specific detection of SNP genotypes. Control samples were available with heterozygous, homozygous and wild-type genotypes of the eight SNPs, which were all detected correctly by the probes. The HRM analysis of the amplicon (expert scanning) could not distinguish homozygous minor from wild-type genotypes for four SNPs (amplicons 15.18, 15.22, 15.24 and 15.27). Of four variants serial dilutions were tested by probe genotyping, which were detectable down to allelic fractions of 6 % (15.18) or 13 % (15.22, 15.23, 15.24) (online resource Supp. Table S2 and Fig. S4).

#### Multiple variants per amplicon

The presence of one heterozygous variant in an amplicon causes heteroduplexes during HRM, altering the melting curve. The effect of an additional second variant in the same amplicon on the melting curve may be less well visible [25]. During our validation, melting curves from six double heterozygous control samples, with a common SNP and additionally a rare variant, showed melting curves well distinguishable from single heterozygotes and wild-types in amplicons 13.1, 13.2, 15.18 and 15.27. Two double heterozygotes were also tested in serial dilutions of the rare variants, in exon 13 and amplicon 15.18. The rare variants were distinguishable down to 6–25 % (online resource Supp. Fig. S4 and S5).

## Discussion

Re-analysis of *APC* in leukocyte DNA by HRM in eligible patients previously tested negative for pathogenic variants by other methods, yielded pathogenic variants in 12 out of 171 (7 %) patients, of whom two appeared to be present as mosaicism. Additionally, in each of two patients with apparently sporadic polyposis, without a variant in leukocyte DNA, a mosaic pathogenic variant was detected in polyp tissue DNA only. The detection of these pathogenic variants facilitates genetic counseling and family testing. The previously used pre-screening techniques SSCP, DGGE and PTT have detected most *APC* variants in the past, including mosaics. However, these methods have been shown to be incapable of detecting all of the variants present, explaining our results [12, 13, 25–29].

Our minimal detectable allelic fraction by HRM was 2–6 % for 91 % of variants, and 13 % for those remaining. HRM has been shown to detect allelic fractions of between 1 and 13 %, significantly better than the 10–25 % reported for Sanger sequencing [14, 26, 30–34]. Sensitivity has been reported to be optimal for small amplicons of around 100 bp. Our amplicon size was 120–400 bp, in order to span the large *APC* gene with a limited amplicon number [14, 35, 36]. Currently, many diagnostic laboratories are using Sanger sequencing without pre-screening for diagnostic gene testing, since it has become cheaper and easier to perform, also for *APC* [1, 20, 37]. Sanger sequencing would have detected the 10 heterozygous variants. However, the mosaics in patients 11 and 12 (5 and 15 % allelic fraction) were missed by standard sequencing analysis. They were only visible for us after carefully scrutinizing the sequence trace, with the current knowledge of an aberrant melting curve suggestive for a mosaic variant. The two mosaics with the lowest allelic fraction (5–6 %) from Hes et al. [12] were also not detectable by Sanger sequencing, but only by DGGE. Because of its superior sensitivity to detect mosaic variants, HRM is a method to consider using for scanning genes with high occurrence of mosaic variants, like *APC*. However, next generation sequencing (NGS) methods may ultimately be the method of choice for mosaic detection in laboratories for which this is feasible. The advantage of NGS is a limit of detection of ≤1 % and immediate identification of the variant [38–40]. Possible disadvantages of NGS in comparison to HRM are the complexity of the method, the expertise needed and purchase costs of the apparatus. HRM is relatively fast, inexpensive and technically less complicated compared to NGS. HRM is also more flexible, with separate analyses per amplicon, while with NGS large amounts of amplicons are pooled together. With HRM it is easier to repeat failed experiments. For a low limit of detection for mosaicism, NGS needs sufficient sequencing depth, making it more expensive. However, the costs for NGS are reducing, and it will probably be more cost-effective (soon and for many laboratories/countries) to optimize an NGS approach instead of HRM.

HRM is a sensitive, inexpensive and convenient diagnostic method, when its limitations are taken into account [25, 41]. It can be readily applied in almost every standard diagnostic laboratory. Known limitations of HRM, like limited sensitivity for particular variant types, such as homozygous and base-pair neutral variants, variants located in nucleotide stretches and multiple variants per amplicon, were also shown in our study [17, 25]. Variants in known nucleotide stretches, like the T^7^A^13^-stretch in intron 3, can be detected by using an unlabeled probe covering the stretch (data not shown). No adaptations were made for homozygous variant detection, as they are considered as embryonically lethal in *APC* [42].

Sensitivity of HRM, and also sequencing, for mosaic variants can be improved by Cold-PCR, down to allelic fractions of 0.1–1 % [43]. Cold-PCR might be challenging to optimize for large scale gene scanning [44]. Cold-PCR applications have mostly been described for pathogenic variant hotspot regions and one for multiplex PCR of the coding region of TP53 [43, 45]. *APC* pathogenic hotspot variants and common C>T transitions might be good candidate locations to start with [12]. Other methods suitable for mosaic detection are ultra-sensitive allele specific methods and deep next generation sequencing [12, 39, 46–50].

Clinical implications of the detection of *APC* mosaicism have been described before [12, 13, 51]. Depending on the timing of occurrence and distribution during embryonic development, a mosaic variant can be present in blood, affected tissue and/or germ cells. The distribution and level of the mosaic variant can indicate its heritability, phenotype and detectability. If a mosaic variant is present in the germ cells, offspring has up to 50 % risk for inheriting the disease. The severity of the phenotype of a heterozygous germline pathogenic *APC* variant is dependent on its position in the gene. Part of described mosaic patients have milder phenotypes compared to heterozygous carriers of similar variants [12, 13]. Our four detected mosaic variants were located at positions related to a severe phenotype, if heterozygous. Patients 11 and 12, with a mosaic variant in both blood and polyps, had a relatively late age of onset, but both had extracolonic features. Patients 13 and 14 had a severe phenotype. However the polyps of patient 13 were limited to the rectosigmoid. None of our four mosaic variants were CGA to TGA transitions, which were previously described to be a significant portion of mosaic variants [12, 13].

Blood leukocyte DNA is most commonly used for testing for pathogenic germ line variants [12, 51, 52]. To demonstrate the value of testing affected tumor tissue for the detection mosaics, and the possibilities of HRM, two cases of whom we had polyp tissue available were analyzed as ‘proof of principle’ (patients 13 and 14). Both patients showed a pathogenic *APC* variant recurrent in multiple polyps, without detectable pathogenic variant in blood. Both patients had a clear polyposis phenotype and early age of onset, without detectable pathogenic germ line variants in the *APC* gene or *MUTYH* gene. Patient 13 had polyps limited to the rectosigmoid, suggestive of mosaicism. Patient 14 had a typical FAP phenotype, for which the chance of detection of pathogenic *APC* or *MUTYH* germ line variants is expected to be very high. Finding the same pathogenic variant recurrent in multiple polyps in each of the two patients (100 % detection ratio of somatic mosaicism), should in this case be seen as a coincidental finding. One comparable mosaic patient was earlier described, with a pathogenic variant present in five analyzed adenomas and not detectable in blood [12]. It is mandatory to build larger series of patients without pathogenic germ line variants in the polyposis genes and collect their polyp material for somatic *APC* variant analysis [12, 13, 51]. For distinguishing between mosaicism in a substantial part of the colon and an isolated somatic variant limited to one tumor, analysis of multiple polyps and/or surrounding normal tissue is necessary.

A significant group of patients with multiple colorectal polyps remains genetically unexplained after extensive testing for pathogenic *APC* and *MUTYH* variants [8, 20]. Yet undetected *APC* (mosaic) variants will probably explain a small minority of cases. Other screening approaches, like next generation sequencing (NGS), for searching genetic, epigenetic or multifactorial aberrations inside or outside *APC* need to be explored [53]. Recently, pathogenic variants in the *POLE* and *POLD1* gene were found to explain a small portion of polyposis cases [9].

In conclusion, rescreening of *APC* by a uniform sensitive detection method like the described HRM method detects heterozygous and mosaic variants previously missed by different conventional methods in group of genetically unexplained patients with multiple colorectal polyps. In addition, scanning *APC* in DNA isolated from multiple independent polyps of two patients successfully detected mosaic pathogenic *APC* variants present in affected tissue, but undetectable in blood. By using HRM and by screening of tumor DNA, we have genetically explained a further small portion of unexplained polyposis patients from our cohort.

## Electronic supplementary material

Below is the link to the electronic supplementary material.

Supplementary material 1 (DOCX 1463 kb)
