# Supplementary material for: High-resolution melting (HRM) re-analysis of a polyposis patients cohort reveals previously undetected heterozygous and mosaic APC gene mutations
Source: Fam Cancer. 2015 Jan 21;14(2):247–57. doi: 10.1007/s10689-015-9780-5 (PMC4430602; doi:10.1007/s10689-015-9780-5)
Supplement: Supplementary file 1 — Supplementary material 1 (DOCX 1463 kb) [file 10689_2015_9780_MOESM1_ESM.docx]

**Online Resource**

**Journal:** Familial Cancer

**Title:** High-resolution melting (HRM) re-analysis of a polyposis patients cohort reveals previously undetected heterozygous and mosaic APC gene mutations

**Authors**: Astrid A. Out, Ivonne J.H.M. van Minderhout, Nienke van der Stoep, Lysette S. R. van Bommel, Irma Kluijt, Cora Aalfs, Marsha Voorendt, Rolf H.A.M. Vossen, Maartje Nielsen, Hans F.A. Vasen, Hans Morreau, Peter Devilee, Carli M.J. Tops, Frederik J. Hes

**Corresponding author:**

Frederik J. Hes

Department of Clinical Genetics, Leiden University Medical Center

P.O. Box 9600

2300 RC Leiden

The Netherlands

f.j.hes@lumc.nl

**Supplementary tables**

Supp. Table S1 Primers and probes

| Amplicon | F/R/Probe | Primer/Probe ^a^ | Bp ^b^ | GC% ^b^ | Annealing  (°C) | Domains ^c^ |
| --- | --- | --- | --- | --- | --- | --- |
|  | M13 F ^b^ | TGTAAAACGACGGCCAGT … |  |  |  |  |
|  | M13 R ^b^ | CAGGAAACAGCTATGACC … |  |  |  |  |
| 1 | F | **…** AAAAACAAGCAGCCACTAAAT | 281 | 35 | 65 | 1 |
|  | R | **…** CAAGTTTACAAGAGGGAATACTGAATA |  |  |  |  |
| 2 | F | TCTCAGCATACTTAAATGTCAAGAAATAC | 239 | 29 | 61 | 1 |
|  | R | CTACACACCTAAAGATGACAATTTG |  |  |  |  |
| 3.1 | F | TTTAGACTGCTTAAAGCAATTGT | 197 | 40 | 61 | 2 ^c^ |
|  | R | CTCTTCTTGGAAATGAACCCATAG |  |  |  |  |
| 3.2 | F | GCCGGGAAGGATCTGTAT | 242 | 36 | 65 | 1 |
|  | R | GCTCTAAGTGTTAGCTATCACC |  |  |  |  |
| 4 | F | TATTGCTCTTCTGCAGTCTTTATTAG | 268 | 29 | 65 | 1 |
|  | R | ATTCAGTTATCCTGAATTTTAATGGATTAC |  |  |  |  |
|  | probe | *GTTTAAACGTACCTTTTTTTAAAAAAAAAAAAATAGGTC* ^d^ |  |  |  |  |
| 5 | F | CATGCACCATGACTGACGTA | 393 | 32 | 61 | 2 |
|  | R | GTTGCTCAGCAGCCATGATA |  |  |  |  |
|  | probe | *CTTTTTTATTATTTGTGGTTTTAGTTTTCCTTACAAACAG* |  |  |  |  |
| 6 | F | AAGTGGTAGCCATAGTATGATTATTTC | 216 | 32 | 65 | 2 |
|  | R | CAGAATAACTACCTATTTTTATACCCACA |  |  |  |  |
| 7 | F | CCTTGGGCTAAGAAAGCCT | 246 | 37 | 65 | 2 ^c^ |
|  | R | ATCATTCTTAGAACCATCTTGCTTCATA |  |  |  |  |
| 8 | F | GAGTACCTTAACATGATGTTATCTGTATT | 224 | 39 | 63 | 2 ^c^ |
|  | R | TAGCAAAGTAGTCATGGCATTAG |  |  |  |  |
| 9.1 | F | TAAATATCCCATTCATCACTTAATTGGTT | 172 | 37 | 63 | 1 |
|  | R | GGAGCTAGACATAGCTAGCAA |  |  |  |  |
| 9.2 | F | GTCAATGCTTGGTACTCATGAT | 226 | 48 | 63 | 1 |
|  | R | CTGTGAGTGAATGATGTTGTGG |  |  |  |  |
| 9.3 | F | CAGTAAAGAGGCTCGGG | 229 | 49 | 63 | 1 |
|  | R | CGCCCGCCGC-CTTTGAAACATGCACTACGAT |  |  |  |  |
| 10 | F | TTTAAAGTACAATAAACATCATTGCTCTTC | 212 | 33 | 65 | 1 |
|  | R | CCACCAGTAATTGTCTATGTCA |  |  |  |  |
| 11 | F | AAGTTACCAACTTGGTACCAG | 271 | 35 | 63 | 2 |
|  | R | TGAAAGTAAATTAACTCATACCTGAGCTA |  |  |  |  |
|  | probe | *CTGTGAAATGTACGGGCTTACTAATGACCAC* |  |  |  |  |
| 12 | F | AAAACTGAATTAGACATTTAGTAGCCA | 255 | 38 | 65 | 2 |
|  | R | AACTGCCCTCTAAGCCT |  |  |  |  |
| 13 | F | AAACAAAAAAGCAACTAGTATGATTTTATG | 294 | 31 | 65 | 1 |
|  | R | ATAGTACTGAATAATACACAGGTAAGAAA |  |  |  |  |
| 13.1 | F | CGGCTAGCCAGAATTTCTTT | 190 | 32 | 63 | 1 |
|  | R | CCAACTTCTCGCAACGTCTT |  |  |  |  |
|  | probe | *GCAGGTTATTGCGAGTGTTTTGAGG* |  |  |  |  |
| 13.2 | F | AGTGTTTTGAGGAATTTGTCTTG | 217 | 34 | 63 | 1 |
|  | R | CTGAATAATACACAGGTAAGAAATTAGGAAA |  |  |  |  |
| 14.1 | F | CAATATCAGTAACATAGAAGTTAATGAGAG | 192 | 38 | 65 | 1 |
|  | R | GAGTGCCAACCAAAAATGC |  |  |  |  |
| 14.2 | F | TTGCACTGAGAATAAAGCTGATATATG | 249 | 36 | 65 | 2 ^c^ |
|  | R | CGCCCGC-ATTATATAAAACATTGCTTACAATTAGGTC |  |  |  |  |
| 15.1 | F | TGTTACTGCATACACATTGTGACC | 202 | 35 | 65 | 1 |
|  | R | CCCATGTCCCATAATGCTTC |  |  |  |  |
| 15.2 | F | GTGGAATCTCTCAGCAAGAAAT | 238 | 44 | 60 | 1 |
|  | R | TTCTGCTTCTAGGGCTTTTTGTT |  |  |  |  |
| 15.3 | F | GGATGCCAATATTATGTCTCCTG | 238 | 39 | 60 | 1 |
|  | R | GCCAGTATTAAAATTGTCTGACCTA |  |  |  |  |
| 15.4 | F | CAAGCAAAGTCTCTATGGTGATTA | 239 | 40 | 60 | 1 |
|  | R | CTGGATTTTCTGTTGCTGGATG |  |  |  |  |
| 15.5 | F | TCGTTCTGAAAAAGATAGAAGTTTGG | 230 | 45 | 65 | 1 |
|  | R | CATTTCTCTCATCTGTCACACAA |  |  |  |  |
| 15.6 | F | GCCATTCATACCTCTCAGGA | 230 | 38 | 65 | 1 |
|  | R | CCATCACTACTACTGACACTATTTAAAC |  |  |  |  |
| 15.7 | F | GACATGTTCTATGCCTTATGCCA | 238 | 36 | 65 | 1 |
|  | R | TGGTGTATCTAGTTCTCCATCATTAT |  |  |  |  |
| 15.8 | F | CAATACCCAGCCGACCTA | 233 | 36 | 65 | 1 |
|  | R | GGATAAGTTGTACTTTGATTCCTTGA |  |  |  |  |
| 15.9 | F | GCAAGACCCAAACACATAATAGA | 232 | 39 | 65 | 1 |
|  | R | GGCTTACATTTTGATTAATTCCATGAT |  |  |  |  |
| 15.10 | F | ATACAGGTCACGGGGAG | 225 | 38 | 65 | 1 |
|  | R | GGCTGATCCACATGACG |  |  |  |  |
| 15.11 | F | ATGAAGAAGAAGAGAGACCAACA | 240 | 37 | 65 | 1 |
|  | R | GATGGAGCTGATTCTGCCT |  |  |  |  |
| 15.12 | F | ACATATGTCTTCAAGCAGTGAG | 222 | 40 | 65 | 2 |
|  | R | GCTGATGACAAAGATGATAATGAAC |  |  |  |  |
| 15.13 | F | CACTTGCAAAGTTTCTTCTAT | 207 | 35 | 65 | 1 |
|  | R | GCTGACCTAGTTCCAATC |  |  |  |  |
| 15.14 | F | GACACAGGAAGCAGATTCTGCTAATA | 230 | 46 | 65 | 1 |
|  | R | GTGTCTGAGCACCACTTT |  |  |  |  |
| 15.15 | F | AATCAGCCAGGCACAAAGCTG | 241 | 47 | 65 | 1 |
|  | R | TCTGGAAGATCACTGGGGCTTA |  |  |  |  |
| 15.16 | F | CAGCTCCGTTCAGAGTGA | 230 | 48 | 65 | 1 |
|  | R | CCCTCTGAACTGCAGCA |  |  |  |  |
| 15.17 | F | AATAAAGCACCTACTGCTGAAA | 116 | 44 | 65 | 1 |
|  | R | GTGGCAAAATGTAATAAAGT |  |  |  |  |
| 15.18 | F | AGAGGGTCCAGGTTCTTC | 126 | 46 | 65 | 1 |
|  | R | CTTTCTGTATAAATGGCTCATCG |  |  |  |  |
|  | probe | *ATGCTGATACTTTATTACATTTTGCCACGGAAAGTAC* |  |  |  |  |
|  | probe | *TCCAGATGGATTTTCTTGTTCAT* |  |  |  |  |
| 15.19 | F | TCCAGATGGATTTTCTTGTTCA | 233 | 37 | 65 | 1 |
|  | R | TTTCAATATCATCATCATCTGAATCATCTA |  |  |  |  |
| 15.20 | F | ATGAAAACCAAGAGAAAGAGGC | 237 | 39 | 65 | 2 |
|  | R | GCAACCTGTTTTGTGATGG |  |  |  |  |
| 15.21 | F | TCCACCTGTGGCAAGGAAA | 227 | 46 | 65 | 1 |
|  | R | CTCCTCTAACTCCTTCTCCAG |  |  |  |  |
| 15.22 | F | CCTATAAACTTTTCCACAGCTACAT | 229 | 43 | 61 | 1 |
|  | R | GAATATCACCTTCCTCTGCTTTATTG |  |  |  |  |
|  | probe | *GTTAGAGGAGGAGCACAGTCAGGTGAATTTG* |  |  |  |  |
| 15.23 | F | CTCAAGGAGGAAAAACCTCAT | 238 | 41 | 65 | 1 |
|  | R | ATAGGTTTTACTGGTGAAGTTGG |  |  |  |  |
|  | probe | *CAAGCATCTGCGTCGTCTTCTGCACCCAA* |  |  |  |  |
| 15.24 | F | CTTCTGCACCCAACAAAAATC | 293 | 39 | 65 | 1 |
|  | R | GTAAGGAGTTCCTTCAATAGGCG |  |  |  |  |
|  | probe | *ATAATTCCAAGGACTTCAATGATAAGCTCCCA* |  |  |  |  |
| 15.25 | F | ATGAAGATAGAGTCAGAGGAAGT | 241 | 38 | 61 | 1 |
|  | R | GTCTTATTAGCTGATTGTTGGTTG |  |  |  |  |
| 15.26 | F | AAAAGAAAATAAGGAATCAGAGGC | 228 | 40 | 65 | 1 |
|  | R | GGAGTATTTTCAATAGCAAAATTCTGTAAC |  |  |  |  |
| 15.27 | F | TTTTCCCCAGTCATCCAAAGAC | 274 | 40 | 65 | 1 |
|  | R | TCTTGAGAAACAAACTGGGGT |  |  |  |  |
|  | probe | *GAAAATACTCCAGTTTGCTTTTCTCATAATTCCTC* |  |  |  |  |
| 15.28 | F | AAACCTCAAGCATCAGGC | 246 | 39 | 61 | 1 |
|  | R | ATCTTTCAAATCAAGTGTCAGAT |  |  |  |  |
| 15.29 | F | GACTCAAGGGTGATAATGAAAAAC | 249 | 39 | 65 | 1 |
|  | R | GGGAAAGGATGGAATCTGAAT |  |  |  |  |
| 15.30 | F | GCAAATTCCATAGTAAGTAGTTTACATCAA | 249 | 38 | 65 | 1 |
|  | R | TCCTTTGATTCCTTTACTTTCAGATTCTA |  |  |  |  |
| 15.31 | F | GCCCACGAATTCTAAAACCAG | 244 | 38 | 65 | 2 |
|  | R | GGACTTGTACTTGAGGAGCTA |  |  |  |  |
| 15.32 | F | CCTTCAATCTCTCGAGGCAG | 247 | 47 | 65 | 1 |
|  | R | ATCCTGATCTAGAAGGTGCTTT |  |  |  |  |
| 15.33 | F | AGAATTAAGCCCTGTTGCC | 247 | 46 | 65 | 1 |
|  | R | TCCAGAACCTGAGGACTTAGT |  |  |  |  |
| 15.34 | F | GTCCTCCTAACAAATTATCTCAACTT | 250 | 40 | 65 | 1 |
|  | R | GACATTCTAGAAAGTTCTACCTTTTTATTG |  |  |  |  |
| 15.35 | F | AGTGAGTCTGCCTCCAAAG | 249 | 39 | 65 | 1 |
|  | R | CCTAGTGGGAGAAGCTGG |  |  |  |  |
| 15.36 | F | AGAAAATTGGAGGAATCTGCT | 247 | 45 | 65 | 1 |
|  | R | GACTTTCAGAATGAGACCGT |  |  |  |  |
| 15.37 | F | CTAATCTCAGTCCCACTATAGAGTATAA | 217 | 43 | 65 | 1 |
|  | R | CTGGATTCTGATGAAGCAGAAAG |  |  |  |  |
| 15.38 | F | ATCCCTTCCTCGAGTAAGC | 246 | 38 | 65 | 1 |
|  | R | GCACCATTTGTAGCACCT |  |  |  |  |
| 15.39 | F | GAACATGGAGAAAAATAAAAGAAAATGAAT | 250 | 40 | 65 | 1 |
|  | R | TTTGGATTTGCCTTTTCTGAAAC |  |  |  |  |
| 15.40 | F | TCCTAGATCTGGAAGATCTCCC | 248 | 43 | 65 | 1 |
|  | R | TCTCTGATACAGGGACAGGAT |  |  |  |  |
| 15.41 | F | TGGATGCCCCTGACCAAA | 243 | 47 | 65 | 1 |
|  | R | CTGGAGTTGGGATCTGAGATG |  |  |  |  |
| 15.42 | F | TTACAACCCAAGCCCTAGGA | 245 | 42 | 65 | 2 ^c^ |
|  | R | GAAACAAAATGTCTATATAGCAGTTGTAAT |  |  |  |  |

a GC clamps are underlined

b To all primers (not probes) M13 tails were attached. Primer sequences, bp and GC% are shown exclusive M13 tails

c If two melting domains and marked with "c", it was possible to analyze each melting domain separately, as explained in online resource Supp. Fig. S1

d This probe was not used for scanning of the patient group

Supp. Table S2 All variants found in the 171 patients ^a^

| Amplicon | Variant DNA | Variant protein | Times found  (MAF) | Earlier method ^b^ | Reported ^c^ | dbSNP  MAF ^d^ |
| --- | --- | --- | --- | --- | --- | --- |
| *Pathogenic* | | |  |  |  |  |
| 6 | c.679del | p.Asp227Thrfs*66 | 1 | NA | - |  |
| 9.3 | c.1180C>T | p.Gln394* | 3 | S- D- | L |  |
| 9.3 | c.1248C>A | p.Tyr416* | 1 | S- | HGMD |  |
| 11 | c.1548+1G>A | Splice donor site | 1 | S- | L |  |
| 14.2 | c.1958+1_1958+2dup | Splice donor site | 1 | D- |  |  |
| 15.1 | c.1972_1975del ^e^ | p.Glu658Thrfs*11 | 1 | P- | References ^c^ |  |
| 15.1 | c.2003del | p.His668Profs*2 | 1 | P- | - |  |
| 15.2 | c.2222dup | p.Asn741Lysfs*15 | 1 | D- P- | - |  |
| *Mosaic* | | |  |  |  |  |
| 15.3 | c.2269C>T (5%) | p.Gln757* | 1 | D- P- | C L |  |
| 15.17 | c.4393_4394dup (15%) | p.Ser1465Argfs*9 | 1 | P- SEQ- ^f^ | L |  |
| *Polyp tissue only* | | |  |  |  |  |
| 15.14 | c.4057G>T | p.Glu1353* | 1 |  | C U |  |
| *Polyp tissue only (outside study group)* ^a^ | | |  |  |  |  |
| 15.19/20 | c.4666dup | p.Thr1556Asnfs*3 | 1 |  | C L U |  |
| *Rare polymorphisms / VUS* | | |  |  |  |  |
| 3.1 | c.295C>T ^e^ | p.Arg99Trp | 1 (0.003) | SEQ+ | L U rs139196838 | 0.001 |
| 5 | c.645+33G>A | p.? | 2 (0.006) | SEQ+ | rs200162147 | 0.001 |
| 6 | c.705A>G | p.= (p.Leu235Leu) | 1 (0.003) | D+, SEQ+ | L U rs147036141 | 0.003 |
| 14.2 | c.1958+8T>C | p.? | 1 (0.003) | S- D- | L rs62626346 | 0.002 |
| 15.4/5 | c.2569G>A | p.Gly857Arg | 1 (0.003) | (P) | - |  |
| 15.8/9 | c.3173A>G | p.Asp1058Gly | 1 (0.003) | (SB) | L rs148725540 | 0.001 |
| 15.10 | c.3386T>C | p.Leu1129Ser | 1 (0.003) | (P) | C L rs143638171 | 0.003-0.007 |
| 15.13/14 | c.3920T>A | p.Ile1307Lys | 1 (0.003) | SEQ+ | C L rs1801155 | 0.002-0.007 |
| 15.22/23 | c.5140G>A | p.Asp1714Asn | 1 (0.003) | (P) | rs148275069 | 0.0001 |
| 15.24 | c.5348C>G | p.Thr1783Ser | 1 (0.003) | (P) | - |  |
| 15.24 | c.5501_5506del | p.Val1834_Arg1835del | 1 (0.003) | (P) | - |  |
| 15.29/30 | c.6363_6365dup | p.Ala2122dup | 1 (0.003) | (P) | - |  |
| 15.33 | c.6921G>A | p.= (p.Ser2307Ser) | 1 (0.003) | NA | L rs2229993 | 0.002-0.008 |
| 15.34 | c.7098T>C | p.= (p.Tyr2366Tyr) | 1 (0.003) | (P) | - |  |
| 15.38 | c.7786T>G | p.Ser2596Ala | 1 (0.003) | (P) | rs138137162 | 0.0001 |
| 15.38 | c.7862C>G | p.Ser2621Cys | 1 (0.003) | SEQ+ | L U rs72541816 | 0.005-0.03 |
| *Common polymorphisms* | | | *het/hom* |  |  |  |
| 2 | c.136-53T>C | p.? | 12/0 (0.035) |  | rs2304793 | 0.009-0.048 |
| 5 | c.645+32C>T | p.? | 36/1 (0.111) |  | L rs2909961 | 0.083-0.093 |
| 11 | c.1458T>C | p.= (p.Tyr486Tyr) | 85/60 (0.600) |  | L rs2229992 | 0.575-0.603 |
| 13.1 | c.1635G>A | p.= (p.Ala545Ala) | 74/68 (0.614) |  | L rs351771 | 0.584-0.642 |
| 13.2 | c.1743+78A>G | p.? | 8/0 (0.023) |  | L rs62364023 | 0.033 |
| 15.16 | c.4326T>A | p.= (p.Pro1442Pro) | 3/0 (0.009) |  | C L U rs67622085 | 0.007-0.017 |
| 15.18 | c.4479G>A | p.= (p.Thr1493Thr) | 75/68 (0.617) |  | L U rs41115 | 0.586-0.642 |
| 15.22 | c.5034G>A | p.= (p.Gly1678Gly) | 73/70 (0.623) |  | L rs42427 | 0.592-0.642 |
| 15.23 | c.5268T>G | p.= (p.Ser1756Ser) | 77/68 (0.623) |  | rs866006 | 0.592-0.634 |
| 15.24 | c.5465T>A | p.Asp1822Val | 62/102 (0.780) |  | L U rs459552 | 0.765-0.796 |
| 15.27 | c.5880G>A | p.= (p.Pro1960Pro) | 72/67 (0.602) |  | L rs465899 | 0.592-0.642 |
| 15.34/35 | c.7201C>T | p.= (p.Leu2401Leu) | 5/0 (0.015) |  | L U rs2229994 | 0.008-0.021 |
| 15.36 | c.7504G>A | p.Gly2502Ser | 7/0 (0.205) |  | L rs2229995 | 0.008-0.097 |

a Of two patients DNA isolated from polyps was available for HRM of *APC*. Patient 13 was part of the study group of 171 patients referred between 1995 and 2007 and patient 14 was referred after this interval in 2008

b Earlier tested by “S” (SSCP), “D” (DGGE), “P” (PTT), “SEQ” (Sanger sequencing), “SB” (Southern blot). “NA”: not analyzed for this particular region. “+”: detectable and “-“: not detectable, by the earlier method. The method was shown between “()” it was not possible to detect the variant by this particular technique

c New variants, not reported elsewhere, are marked with “-“. Variants reported elsewhere, in independent patients, are marked with “C” (COSMIC database), the “rs” number (dbSNP), “L” (LOVD), “U” (UMD), or references [20,22]

d dbSNP build 138 allele frequency in Caucasian populations (http://www.ncbi.nlm.nih.gov/SNP/, [23]

e c.295C>T (VUS) and c.1972_1975del (pathogenic) were both detected in one patient (patient 8 in Table 2)

f Visible after revision of the sequence after the positive melting curve was found (Fig. 1)

Supp. Table S3 Validated variant types and allelic fraction detection limits ^a^

|  |  | Detectable down to | | | |
| --- | --- | --- | --- | --- | --- |
| Type | # | 2% | 3% | 6% | 13% |
| A<>C or G<>T | 10 | 1 (10%) | 6 (60%) | 3 (30%) |  |
| A<>G or C<>T | 40 | 3 (8%) | 6 (15%) | 30 (75 %) | 1 (3%) |
| A<>T | 7 |  | 1 (14%) | 4 (57%) | 2 (29%) |
| C<>G | 3 |  |  | 3 (100%) |  |
| del 1 bp | 16 | 1 (6%) | 3 (19%) | 9 (56%) | 3 (19%) |
| del 2-5 bp | 18 |  | 3 (17%) | 15 (83%) |  |
| del >5 bp | 2 |  |  | 2 (100%) |  |
| ins 1 bp | 14 |  | 4 (29%) | 5 (36%) | 5 (36%) |
| ins 2-5 bp | 3 | 1 (33%) | 1 (33%) | 1 (33%) |  |
| ins >5 bp | 3 |  | 2 (67%) | 1 (33%) |  |
| All | 116 | 6 (5%) | 26 (22%) | 73 (63%) | 11 (9%) |
| cumulative % |  | 5% | 28% | 91% | 100% |

a Variants were grouped for type of base-pair change or according to number of deleted or inserted nucleotides. Per type of variant the total number of tested unique variants and is shown and the portions of variants detectable down to each tested allelic fraction detection limit

**Supplementary figures**


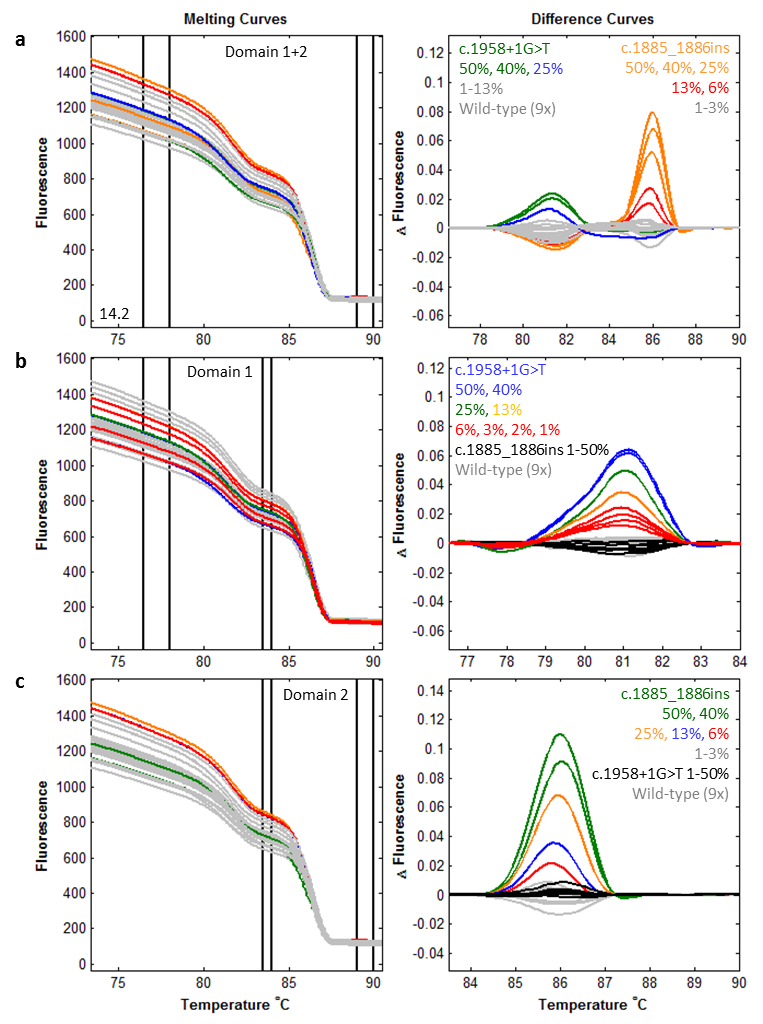


Supp. Fig. S1 Example of melting curves of an amplicon with two melting domains (amplicon 14.2). a. detection levels when normalized at the start of the melting curve (near 100% fluorescence) and at the end of the melting curve (near 0% fluorescence), analyzing domains 1 and 2 together. b. normalization directed at domain 1. c. normalization directed at domain 2. It must be noted that application of such settings did not work as well for all of the amplicons with two melting domains. Some amplicons needed to be redesigned or split in two (data not shown). Melting curves with more than two melting domains are not suitable to analyze (data not shown)


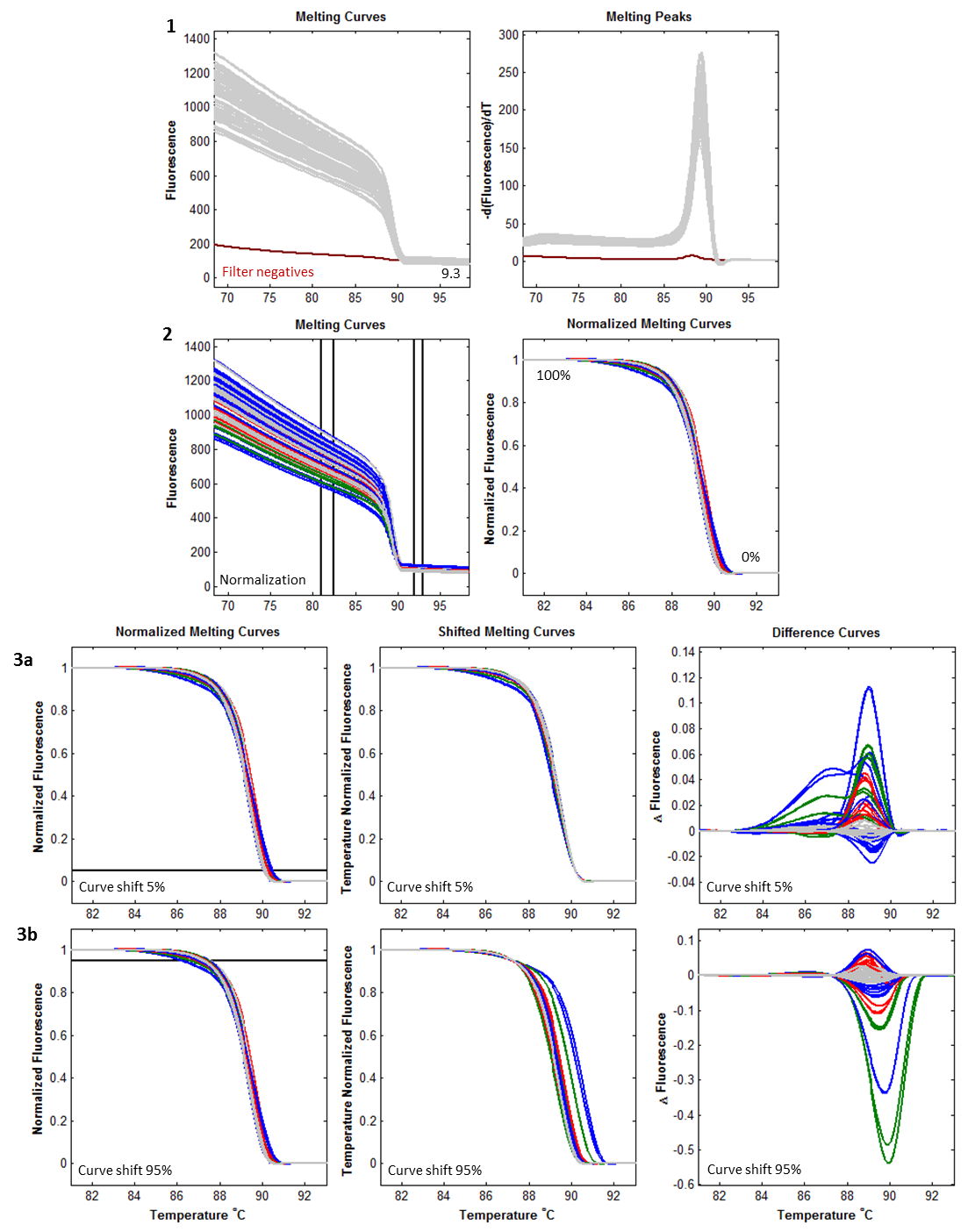


Supp. Fig. S2 Analysis steps and settings in Lightscanner melting curve analysis: 1. Filtering out negatives, 2. Normalizing curves at 100% and 0% fluorescences for fluorescence intensity differences between samples, 3. Temperature shifting correction for temperature differences across the PCR plate (a. at 5% fluorescence, b. at 95% fluorescence). Difference plot of melting speed were used for final analysis of melting curve differences (an analysis called “expert scanning” in the Lightscanner software)


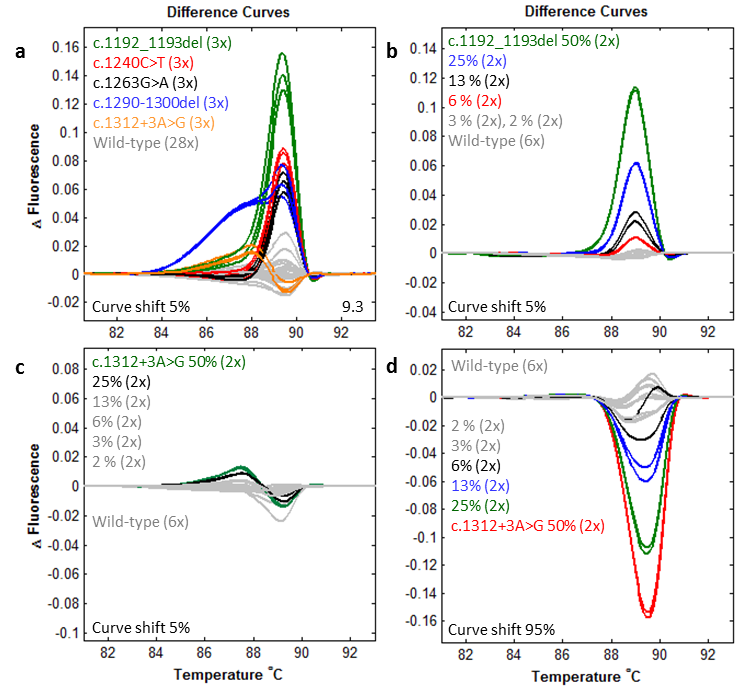


Supp. Fig. S3 Differences in detectability between different heterozygous variants and their serial dilutions. a. Different heterozygous variants in amplicon 9.3. Heterozygous variants with a difference curve with a low amplitude were generally detectable down to higher minimal allelic fractions compared to variants with high amplitude (e.g. c.1192_1193del versus c.1312+3A>G, shown in b, c). d. using curve shift setting 95%, improved results for variant c.1312+3A>G, from 25% to 13% detected dilution


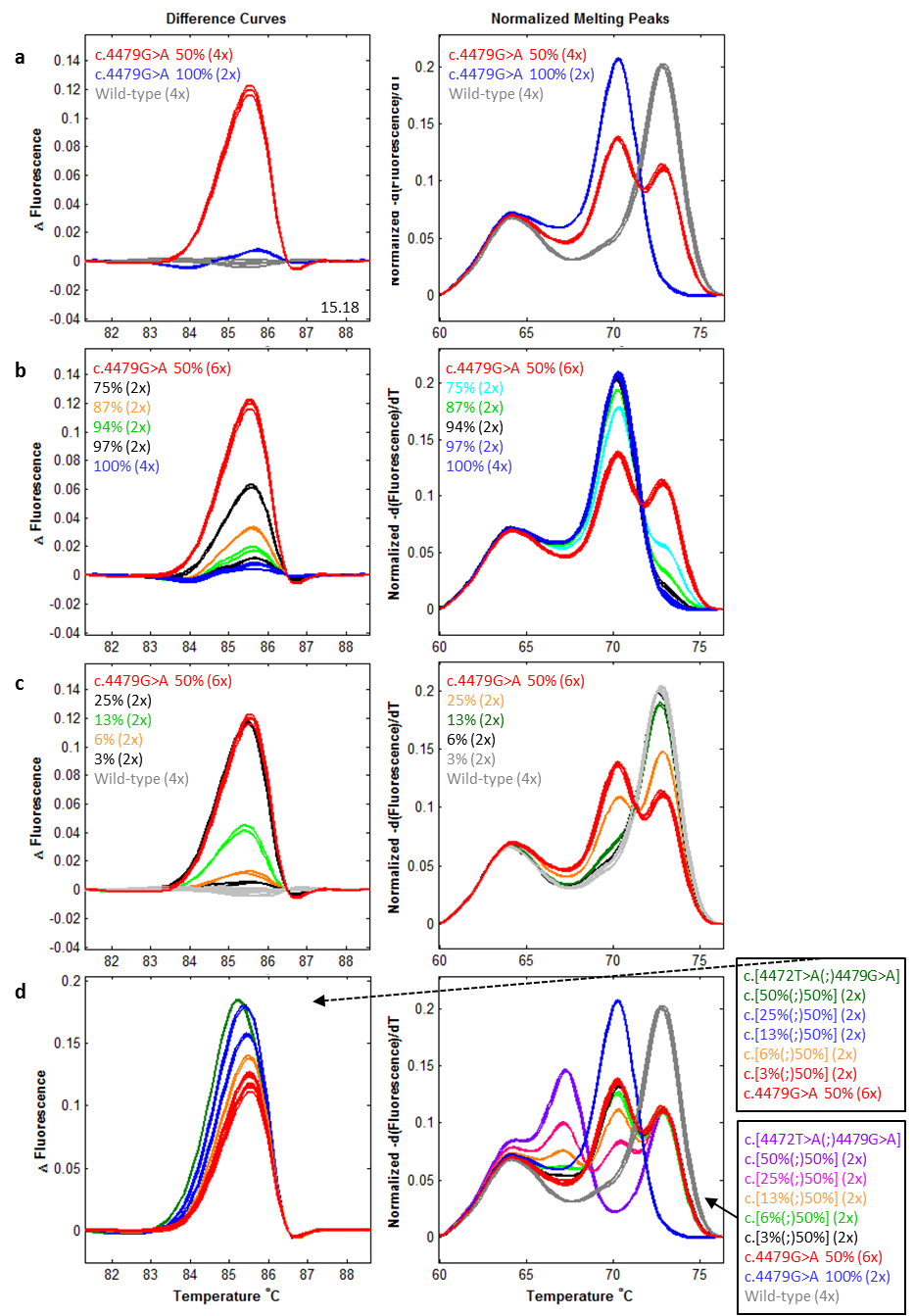


Supp. Fig. S4 Detectability of low allelic fractions by probe genotyping and expert scanning, including detection of two variants in one sample. a. Expert scanning and unlabeled probe genotyping of control samples with homozygous, heterozygous and wild-type genotypes of the common SNP c.4479G>A in the 126 bp amplicon 15.18. b-c. Serial dilutions of a c.4479G>A heterozygous sample, with a homozygous sample and with a wild-type sample, respectively. Expert scanning shows more sensitive detection compared to probe genotyping for small allelic fraction differences. d. Detection of multiple variants in one sample. The sample contained the common SNP and the variant c.4472T>A, both located under the same probe*. In serial dilutions (with DNA heterozygous for the common SNP), c.4472T>A was detectable down to 6% by expert scanning and probe genotyping. The 3% curve in probe genotyping is shown in black, but was hardly distinguishable from samples with only the common SNP.

*For amplicon 15.18 two adjacent probes were designed, covering 60 bp in total. One probe covered the common SNP and one probe covered the area next to the SNP, to add possible sensitivity. However no variants were found in the area of the second probe (online resource Supp. Table S1)


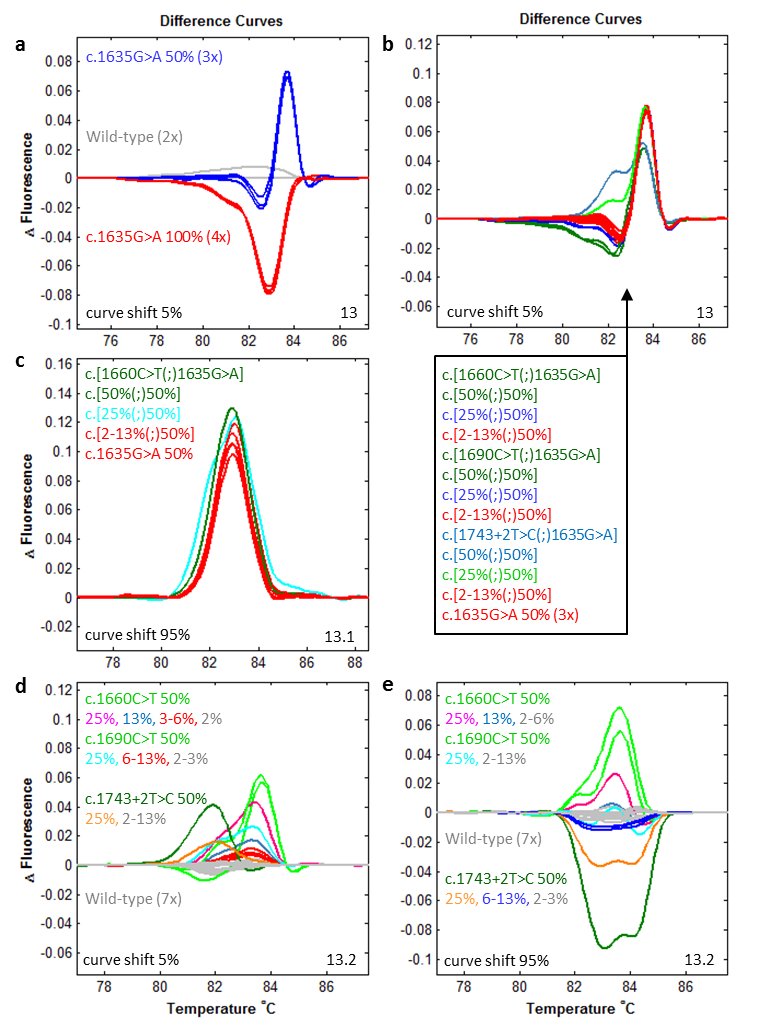


Supp. Fig. S5 Detectability of multiple variants per sample in one amplicon. a. Control samples with homozygous, heterozygous and wild-type genotypes of the common SNP c.1635G>A in the 294 bp exon 13 amplicon. b. Three samples, each heterozygous for c.1635G>A and each with a unique additional variant in exon 13 (c.1660C>T, c.1690C>T and c.1743+2T>C). In serial dilutions (with DNA heterozygous for the common SNP) the lowest detectable allelic fraction for all three variants was 25%. c-e. Detection of the same three variants in exon 13 after splitting the amplicon into two amplicons, one with (13.1, 190 bp) and one without the common SNP (13.2, 217 bp). In amplicon 13.2 the three rare variants were detectable down to 6.3%. In amplicon 13.1 one rare variant was located and detectable down to 25%, like in the larger exon 13 amplicon
